# Association of inflammatory markers with cerebral small vessel disease in community-based population

**DOI:** 10.1186/s12974-022-02468-0

**Published:** 2022-05-06

**Authors:** Lingling Jiang, Xueli Cai, Dongxiao Yao, Jing Jing, Lerong Mei, Yingying Yang, Shan Li, Aoming Jin, Xia Meng, Hao Li, Tiemin Wei, Yongjun Wang, Yuesong Pan, Yilong Wang

**Affiliations:** 1 Department of Neurology, Beijing Tiantan Hospital, Capital Medical University, No.119 South 4th Ring West Road, Fengtai District, Beijing, 100070 China; 2 China National Clinical Research Center for Neurological Diseases, Beijing, 100070 China; 3 Department of Neurology, Lishui Hospital, Zhejiang University School of Medicine, Lishui, 323000 China; 4 Cerebrovascular Research Lab, Lishui Hospital, Zhejiang University School of Medicine, Lishui, 323000 China; 5 Department of Cardiology, Lishui Hospital, Zhejiang University School of Medicine, Lishui, 323000 China

**Keywords:** Cerebral small vessel disease, Inflammation, Neutrophil count, Neutrophil–lymphocyte ratio, Systemic immune-inflammation index

## Abstract

**Background:**

This study investigated the relationships of neutrophil count (NC), neutrophil-to-lymphocyte ratio (NLR) and systemic immune-inflammation index (SII) with cerebral small vessel disease (CSVD).

**Methods:**

A total of 3052 community-dwelling residents from the Poly-vasculaR Evaluation for Cognitive Impairment and vaScular Events (PRECISE) study were involved in this cross-sectional study. CSVD burden and imaging markers, including white matter hyperintensity (WMH), lacunes, cerebral microbleeds (CMBs) and enlarged perivascular spaces in basal ganglia (BG-EPVS), were assessed according to total CSVD burden score. The associations of NC, NLR and SII with CSVD and imaging markers were evaluated using logistic regression models. Furthermore, two-sample Mendelian randomization (MR) analysis was performed to investigate the genetically predicted effect of NC on CSVD. The prognostic performances of NC, NLR and SII for the presence of CSVD were assessed.

**Results:**

At baseline, the mean age was 61.2 ± 6.7 years, and 53.5% of the participants were female. Higher NC was suggestively associated with increased total CSVD burden and modified total CSVD burden (Q4 vs. Q1: common odds ratio (cOR) 1.33, 95% CI 1.05–1.70; cOR 1.28, 95% CI 1.02–1.60) and marginally correlated with the presence of CSVD (OR 1.29, 95% CI 1.00–1.66). Furthermore, elevated NC was linked to a higher risk of lacune (OR 2.13, 95% CI 1.25–3.62) and moderate-to-severe BG-EPVS (OR 1.67, 95% CI 1.14–2.44). A greater NLR was related to moderate-to-severe BG-EPVS (OR 1.68, 95% CI 1.16–2.45). Individuals with a higher SII had an increased risk of modified WMH burden (OR 1.35, 95% CI 1.08–1.69) and moderate-to-severe BG-EPVS (OR 1.70, 95% CI 1.20–2.41). MR analysis showed that genetically predicted higher NC was associated with an increased risk of lacunar stroke (OR 1.20, 95% CI 1.04–1.39) and small vessel stroke (OR 1.21, 95% CI 1.06–1.38). The addition of NC to the basic model with traditional risk factors improved the predictive ability for the presence of CSVD, as validated by the net reclassification index and integrated discrimination index (all *p* < 0.05).

**Conclusions:**

This community-based population study found a suggestive association between NC and CSVD, especially for BG-EPVS and lacune, and provided evidence supporting the prognostic significance of NC.

**Supplementary Information:**

The online version contains supplementary material available at 10.1186/s12974-022-02468-0.

## Background

Cerebral small vessel disease (CSVD) is the most common pathological neurological process and it has an essential role in causing stroke and dementia [1]. Inflammation plays a critical role in the pathophysiology of CSVD. Systemic inflammation processes are thought to be closely associated with endothelial dysfunction, blood–brain barrier permeability and cerebral blood flow autoregulation, which may thus influence the development of CSVD [2]. Studies have consecutively shown a close association between alterations in biomarkers of inflammation and CSVD [3, 4]. The easy access of blood samples provides convenience for investigating the peripheral inflammation in disease. Emerging evidence has indicated that components of the hematological inflammatory system, especially neutrophils, can infiltrate and accumulate in the perivascular space, resulting in blood–brain barrier disruption and vascular diseases [5, 6]. The neutrophil-to-lymphocyte ratio (NLR) and systemic immune-inflammation index (SII) derived from blood cell counts can suitably reflect the immune status [7, 8]. Increased NLR and SII were found to be correlated with a deterioration of vascular diseases and are powerful prognostic indicators of these diseases [9, 10].

To the best of our knowledge, the correlations of neutrophil count (NC), NLR and SII with CSVD have not been adequately investigated. Current reports indicated that elevated NLR was associated with the volume of white matter hyperintensity (WMH) in a healthy population and with the white matter microstructure in Parkinson's disease, while SII was related to white matter microstructure in COVID-19 survivors [11–13]. Nevertheless, the relationships of NC, NLR and SII with the presence and severity of CSVD and imaging phenotypes remain unknown.

In this study, we evaluated the associations of NC, NLR and SII with the presence of CSVD, CSVD burden and imaging markers, in a relatively large community population cohort. Furthermore, we conducted Mendelian randomization (MR) analysis, which can avoid potential unmeasured confounders and reverse causation by using genetic variants as instrumental variables, to verify the robustness of the association between NC and CSVD. We explored the predictive performance of adding NC, NLR and SII to the basic model, which includes traditional vascular risk factors.

## Methods

### Study participants

The participants were from the Poly-vasculaR Evaluation for Cognitive Impairment and vaScular Events (PRECISE) study. Details of the rationale and design of the PRECISE study have been described previously [14]. Briefly, the PRECISE study was a population-based prospective cohort study with a comprehensive evaluation of multi-territorial artery stenosis and plaque using advanced vascular imaging techniques and a prospective collection of vascular events and cognitive assessments. This study initially screened 4202 community-dwelling residents aged 50–75 years between May 2017 and September 2019 from six villages and four communities of Lishui city in southeastern China. Among of them, 3433 subjects consented to participate in the study, and 3067 subjects were eligible and enrolled at baseline. The villages and communities selected were living communities with stable populations and little migration to ensure population representativeness. Subjects included in the PRECISE study had a similar sex and age distribution as those excluded and as data from surveys of a nationwide sample.

Participants who had contraindications to magnetic resonance imaging (MRI), life expectancy ≤ 4 years due to advanced cancers or other diseases, and mental diseases were excluded. Subjects with available data from laboratory tests and MRI information were included in this analysis. This study was approved by the ethics committees of both Beijing Tiantan Hospital (IRB approval number: KY2017-010-01) and Lishui Hospital (IRB approval number: 2016-42). Informed consent was obtained from all subjects enrolled in the PRECISE study.

### Baseline clinical assessment

Baseline subject demographics, risk factors (blood pressure, body mass index, drinking and smoking status), medical history (hypertension, diabetes, dyslipidemia and heart disease) and medication use (antihypertensive, lipid-lowing, antidiabetic, antiplatelet, anticoagulant) were collected. Fasting venous blood samples were collected at baseline. Several laboratory tests were conducted, including routine blood examination, total cholesterol, triglycerides, high-density lipoprotein, low-density lipoprotein, fasting blood glucose, and homocysteine levels. All of the data were evaluated through face-to-face interviews by centralized trained personnel at Lishui Hospital.

### Blood cell count assessment

Fasting blood samples were collected during the face-to-face interviews and were sent to the laboratory. Neutrophil, lymphocyte, and platelet counts were detected with flow cytometry (XE-2100, SYSMES, Kobe, Japan). NLR (neutrophil count/lymphocyte count) and SII (platelet count × neutrophil count/lymphocyte count) were calculated with absolute neutrophil count (× 10^9^/L), lymphocyte count (× 10^9^/L) and platelet count (× 10^9^/L) [11, 13].

### MRI acquisition and assessment

Participants underwent brain MRI scans by a 3.0T MRI scanner (Ingenia 3.0T, Philips, Best, The Netherlands) based on a standardized protocol. The MRI sequences included three-dimensional T1-weighted magnetization prepared rapid acquisition gradient-echo (3D T1w MPRAGE), axial T2-weighted, fluid-attenuated inversion recovery (FLAIR), and axial susceptibility-weighted imaging. The detailed scanner parameters are listed in Additional file 1: Table S1. Imaging data were collected in digital imaging and communications in medicine (DICOM) format on discs and further analyzed by the imaging research centre at Beijing Tiantan Hospital. WMH was defined as increased brightness on T2 images in the brain white matter. The periventricular and deep WMH were evaluated according to the Fazekas rating scale [15]. Lacune was defined as a rounded or ovoid lesion of CSF signal measuring 3–20 mm in diameter. Cerebral microbleeds (CMBs) were rounded, hypodense lesions with sizes of 2–10 mm in a gradient-recalled echo image or susceptibility-weighted image. The total number of lacunes and CMBs were also recorded. Enlarged perivascular spaces (EPVS) was defined as small (< 3 mm) punctate or linear hyperintensities on T2 images, and EPVS in the basal ganglia was graded with the semi-quantitative rating scale developed by the Edinburg group [16]. Imaging assessment of each CSVD marker was rated by two well-trained raters (M Zhou, Y Chen, J Pi, and M Zhao, one rater was responsible for two markers) who were blinded to the participants’ clinical data, according to the standards for reporting vascular changes on neuroimaging (STRIVE) [17]. Images with inconsistent results were finally assessed by another senior neurologist (Y Yang) who was blinded to the initial results. The kappa coefficients of CSVD markers on brain MRI between raters were as follows: 0.82 for the Fazekas scale of WMH, 0.80 for the presence of lacune, 0.80 for the presence of CMB and 0.90 for the severity of BG-EPVS.

Based on a previously described and validated total CSVD score designed by Wardlaw’s group, we rated the total CSVD burden on an ordinal scale from 0 to 4. One point was allocated for WMH burden (PV-WMH Fazekas 3 or deep-WMH Fazekas 2–3), presence of lacune, presence of CMB, and moderate-to-severe basal ganglia EPVS (BG-EPVS) (*N* > 10) [18]. Furthermore, we also evaluated the modified total CSVD burden using a recently validated ordinal score designed by Rothwell’s group, which ranges from 0 to 6 [19]. One point was allocated for the presence of lacunes, CMB burden (*N* 1–4), severe BG-EPVS (*N* > 20), modified WMH burden (total periventricular + subcortical WMH grade 3–4), two points were allocated for CMB burden (*N* ≥ 5) and modified WMH burden (total periventricular + subcortical WMH grade 5–6).

### Mendelian randomization study design

We further designed a two-sample MR approach to estimate the effect of NC on CSVD including WMH volume, lacunar stroke, small vessel stroke, any CMBs, mixed CMBs, and lobar CMBs. MR analysis can be used for unbiased detection of causal effects of risk factors on diseases because the genetic variations are allocated randomly at conception. The genetic variants used in an MR design must meet three assumptions: (1) the genetic variants used as instrumental variables are strongly associated with the risk factor of interest (NC); (2) the instrumental variable should not be associated with other confounders; and (3) the instrumental variable is associated with the disease (CSVD) only through the investigated risk factor (NC). Summary-level data on the associations of genetic variants with NC and CSVD were obtained from recently published genome-wide association studies (GWAS) [20–24]. Appropriate ethical approval and patient informed consent were obtained from the original studies. Genetic variants associated with NC were selected as instrumental variables for the MR analysis. Data on the exposure (NC) were derived from a GWAS of up to 408,112 individuals [20], while data on the outcome (CSVD phenotypes) were obtained from GWASs of up to 254,959 individuals [21–24]. The characteristics of these GWASs are presented in Additional file 1: Table S2. Analyses of all phenotypes were overwhelmingly based on subjects of European ancestry.

### Statistical analysis

Categorical variables are presented as frequencies and percentages, while continuous variables are presented as the mean with standard deviation or median with interquartile range. The baseline characteristics of the participants were compared between the absence of CSVD (total CSVD burden = 0) and the presence of CSVD (total CSVD burden ≥ 1) according to the total CSVD score designed by Wardlaw et al. and Rothwell et al., respectively. The Chi-square test was used for categorical variables (such as sex and medical history), and ANOVA or the Kruskal–Wallis test was used for continuous variables (such as age and neutrophil count).

The relationships of the neutrophils, NLR or SII with the presence and severity of CSVD or CSVD image markers were evaluated. For the total CSVD burden, modified total CSVD burden, modified WMH burden and CMB burden, ordinal logistic regression models were conducted and common odds ratios (cORs) with their 95% confidence intervals (CIs) were calculated. For the presence of CSVD and other CSVD image markers, logistic regression models were performed and odds ratios (ORs) with their 95% CIs are presented. Two models were conducted for each outcome. In Model 1, covariates including age and sex were adjusted. In Model 2, age, sex, body mass index, diabetes, hypertension, total cholesterol, high-density lipoprotein, low-density lipoprotein, fasting blood glucose, homocysteine, previous dyslipidemia, previous heart disease, current smoking, current drinking, previous antiplatelet, anticoagulant, antihypertensive, antidiabetic, and lipid-lowering drug use were adjusted. The hypertension adjusted in Model 2 was defined as either self-reported hypertension previously diagnosed by a physician or current use of antihypertensive drugs or systolic blood pressure ≥ 140 mm Hg or diastolic blood pressure ≥ 90 mm Hg. The diabetes adjusted in Model 2 was defined as a self-reported diabetes previously diagnosed by a physician or current use of antidiabetic drugs of fasting plasma glucose ≥ 7.0 mmol/L or 2-h postload glucose ≥ 11.1 mmol/L or hemoglobin A1c ≥ 6.5%.

For all analyses, correction for multiple comparisons was done by using the false discovery rate (FDR) approach, and the result was presented as P_FDR. Statistical significance was set at a P_FDR < 0.05. Association not reaching this threshold, but showing a *p* < 0.05, was considered suggestive of an association.

Moreover, two-sample MR approaches were used to evaluate the association of NC with CSVD using summary-level data of the SNP-NC and SNP-CSVD associations. The random-effect inverse-variance weighted (IVW) is the most widely applied method for MR analysis because it provides robust causal estimates under absence of directional pleiotropy [25]. Here, IVW was performed to estimate the effect by generalized weighted linear regression of SNP-CSVD against SNP-NC estimates with the inverse-variance of SNP-CSVD estimate as weights and the intercept set to zero. Based on summary statistics for each trait, we extracted variants with *r*^2^ < 0.001 (linkage disequilibrium), *p* < 5 × 10^–8^ (genome-wide significance) and minor allele frequency > 0.05. The correction for multiple testing was done for each outcome with FDR. The corrected P_FDR < 0.05 was considered as statistically significant. For heterogeneity assessment of each instrument in the IVW analysis, Cochran’s Q statistic was used (*p* < 0.10 indicates the presence of nominal heterogeneity) [26]. In sensitivity analysis, we further applied four alternative MR methods that were more robust to the inclusion of pleiotropic and/or invalid instruments, including MR-Egger regression, weighted median, Mendelian Randomization Pleiotropy RESidual Sum and Outlier (MR PRESSO) and weighted mode methods. As a measure of pleiotropy of included genetic variants (*p* < 0.05 indicates statistical significance), MR-Egger allows for the estimation of an intercept term which used to represent the average pleiotropic effects of all SNPs [27]. The weighted median method allows the use of invalid instruments under the assumption that at least 50% of the instruments used in the MR analysis are valid [28]. MR PRESSO allows to detect and correct for horizontal pleiotropic outlier SNPs in multi-instrument summary-level MR testing [29]. The weighted mode method assumes a plurality of genetic variants are valid instruments, and has low bias [30]. The effect estimates of genetically predicted NC on CSVD phenotypes were presented as odds ratios (ORs) with their 95% CIs per 1-standard deviation (SD) increment of NC.

Additionally, the net reclassification index (NRI) and absolute integrated discrimination improvement (IDI) were calculated to establish the performance of the addition of NC, NLR or SII to the basic model. The covariates involved in the basic model are the traditional vascular risk factors associated with cerebral small vessel disease, including age, sex, body mass index, diabetes, hypertension, total cholesterol, high-density lipoprotein, low-density lipoprotein, fasting blood glucose, homocysteine, previous dyslipidemia, previous heart disease, current smoking, current drinking, previous antiplatelet, anticoagulant, antihypertensive, antidiabetic, and lipid-lowering drug use. The NRI is used to quantify the amount of correct reclassification introduced by using a model with added variables, while the IDI is used to quantify the increase in the separation of events and nonevents. NRI or IDI > 0 reflects improvements in performance between new and old models [31, 32].

All analyses were performed using R 4.0.3 (R Development Core Team) and SAS software version 9.4 (SAS Institute Inc, Cary, NC).

## Results

### Baseline characteristics

Among all 3067 adult residents enrolled in the PRECISE study, 11 subjects were excluded because of missing laboratory test data and 4 individuals were excluded due to motion artifacts or missing MRI sequences. Thus, 3052 participants were included in this analysis (Additional file 1: Fig. S1). The mean age of the study subjects was 61.2 ± 6.7 years, and 1633 (53.5%) individuals were female. Based on the total CSVD score, 931 (30.5%) participants presented with CSVD, while 1267 (41.5%) subjects presented with CSVD according to the modified total CSVD score. The participants who presented with CSVD were older, male and had a higher prevalence of hypertension, diabetes and heart disease; higher white blood cell count, NC, NLR, SII, low-density lipoprotein, fasting blood glucose and homocysteine; and a higher proportion of medicine use than those without CSVD (Table 1).

**Table 1:** Demographic and clinical characteristics according to CSVD burden

Variables	CSVD (Wardlaw)*			CSVD (Rothwell)^†^		
	Absence of CSVD (*n* = 2121)	Presence of CSVD (*n* = 931)	*P* value	Absence of CSVD (*n* = 1785)	Presence of CSVD (*n* = 1267)	*P* value
Demographic data						
Age, mean ± SD	59.8 ± 6.1	64.6 ± 6.7	< 0.001	59.4 ± 6.1	63.7 ± 6.7	< 0.001
Male, *n* (%)	936 (44.1)	483 (51.9)	< 0.001	812 (45.5)	607 (47.9)	0.19
Risk factors, *n* (%)						
Hypertension	769 (36.3)	545 (58.5)	< 0.001	607 (34.0)	707 (55.8)	< 0.001
Diabetes	409 (19.3)	250 (26.9)	< 0.001	328 (18.4)	331 (26.1)	< 0.001
History of dyslipidemia	412 (19.4)	200 (21.5)	0.19	364 (20.4)	248 (19.6)	0.58
History of heart disease	149 (7.0)	100 (10.7)	0.001	121 (6.8)	128 (10.1)	0.001
Current smoking	439 (20.7)	184 (19.8)	0.56	381 (21.3)	242 (19.1)	0.13
Current drinking	382 (18.0)	189 (20.3)	0.14	324 (18.2)	247 (19.5)	0.35
BMI, median (IQR) (kg/m^2^)	23.5 (21.7–25.6)	23.7 (21.6–25.8)	0.41	23.5 (21.7–25.6)	23.7 (21.6–25.8)	0.35
Laboratory data, median (IQR)						
Hemoglobin, g/L	141.0 (132.0–151.0)	141.0 (133.0–151.0)	0.70	141.0 (132.0–151.0)	141.0 (132.0–151.0)	0.30
White blood cell count (× 10^9^/L^)^)	5.9 (5.1–7.0)	6.2 (5.2–7.2)	< 0.001	5.9 (5.1–7.0)	6.2 (5.2–7.1)	0.002
Neutrophil count (× 10^9^/L)	3.3 (2.7–4.1)	3.5 (2.8–4.4)	< 0.001	3.3 (2.7–4.1)	3.5 (2.8–4.3)	< 0.001
Lymphocyte count (× 10^9^/L)	2.0 (1.6–2.4)	2.0 (1.6–2.4)	0.60	2.0 (1.6–2.4)	2.0 (1.6–2.4)	0.45
Platelet count (× 10^9^/L)	208.0 (176.0–243.0)	206.0 (171.0–241.0)	0.29	207.0 (176.0–242.0)	207.0 (173.0–242.0)	0.54
NLR	1.65 (1.29–2.14)	1.74 (1.33–2.29)	0.001	1.65 (1.28–2.14)	1.72 (1.34–2.25)	0.001
PLR	102.9 (82.9–127.4)	100.0 (80.6–127.1)	0.42	102.6(82.5–127.3)	102.3 (81.6–127.8)	0.94
SII (× 10^9^/L)	340.2 (253.2–459.2)	353.0 (261.0–490.7)	0.03	337.0 (252.6–455.9)	353.4 (258.4–488.5)	0.02
Total cholesterol (mmol/L)	5.24 (4.64–5.90)	5.21 (4.54–5.92)	0.26	5.25 (4.64–5.90)	5.21 (4.56–5.92)	0.19
Triglycerides (mmol/L)	1.44 (1.03–2.13)	1.50 (1.08–2.15)	0.18	1.45 (1.03–2.13)	1.48 (1.06–2.15)	0.29
HDL (mmol/L)	1.33 (1.14–1.57)	1.32 (1.11–1.54)	0.16	1.33 (1.14–1.57)	1.32 (1.12–1.56)	0.31
LDL (mmol/L)	2.75 (2.27–3.28)	2.70 (2.16–3.29)	0.043	2.76 (2.27–3.27)	2.71 (2.23–3.30)	0.25
FBG (mmol/L)	5.53 (5.19–6.04)	5.68 (5.29–6.30)	< 0.001	5.53 (5.19–6.03)	5.64 (5.26–6.26)	< 0.001
HCY (mmol/L)	10.5 (8.8–12.8)	11.3 (9.4–14.2)	< 0.001	10.5 (8.8–12.7)	11.1 (9.2–13.8)	< 0.001
Concomitant medication, n (%)						
Antihypertensive	449 (21.2)	370 (39.7)	< 0.001	345 (19.3)	474 (37.4)	< 0.001
Lipid-lowing	68 (3.2)	51 (5.5)	0.003	54 (3.0)	65 (5.1)	0.003
Antidiabetic	157 (7.4)	117 (12.6)	< 0.001	122 (6.8)	152 (12.0)	< 0.001
Antiplatelet	40 (1.9)	40 (4.3)	< 0.001	29 (1.6)	51 (4.0)	< 0.001
Anticoagulant	1 (0.05)	3 (0.32)	0.16	1 (0.06)	3 (0.24)	0.39

*CSVD* cerebral small vessel disease, *BMI* body mass index, *IQR* interquartile range, *NLR* neutrophil-to-lymphocyte ratio; PLR, platelet-to-lymphocyte ratio; *SII* systemic immune-inflammation index (platelet count × neutrophil count/lymphocyte count); *HDL* high-density lipoprotein, *LDL* low-density lipoprotein, *FBG* fasting blood glucose, *HCY* homocysteine

*Wardlaw: 1 point allocated for presence of lacunes, microbleeds, moderate-to-severe (> 10) perivascular space in basal ganglia, periventricular white matter hyperintensities Fazekas 3 or deep white matter hyperintensities Fazekas 2–3

^†^Rothwell: 1 point allocated for presence of lacunes, 1–4 microbleeds, frequent to severe (> 20) perivascular space in basal ganglia, moderate white matter hyperintensities (total periventricular + subcortical white matter hyperintensities grade 3–4), 2 points allocated for ≥ 5 microbleeds and severe white matter hyperintensities (total periventricular + subcortical white matter hyperintensities grade 5–6)

### Association of NC, NLR, and SII with CSVD

The distribution of the CSVD burden score by quartiles of NC, NLR and SII among all of the included individuals is shown in Additional file 1: Fig. S2. The proportion of presence of CSVD (Wardlaw, total CSVD burden scored 1–4) in the 1st, 2nd, 3rd and 4th quartiles of NC was 27.1%, 27.0%, 32.4% and 35.3%, and the presence of CSVD (Rothwell, total CSVD burden scored 1–6) was 38.7%, 37.5%, 44.8% and 44.9%, respectively. The proportions of the presence of CSVD in the quartiles of NLR or SII increased from 1st to 4th quartiles, similar to NC group.

**Fig. 1:**
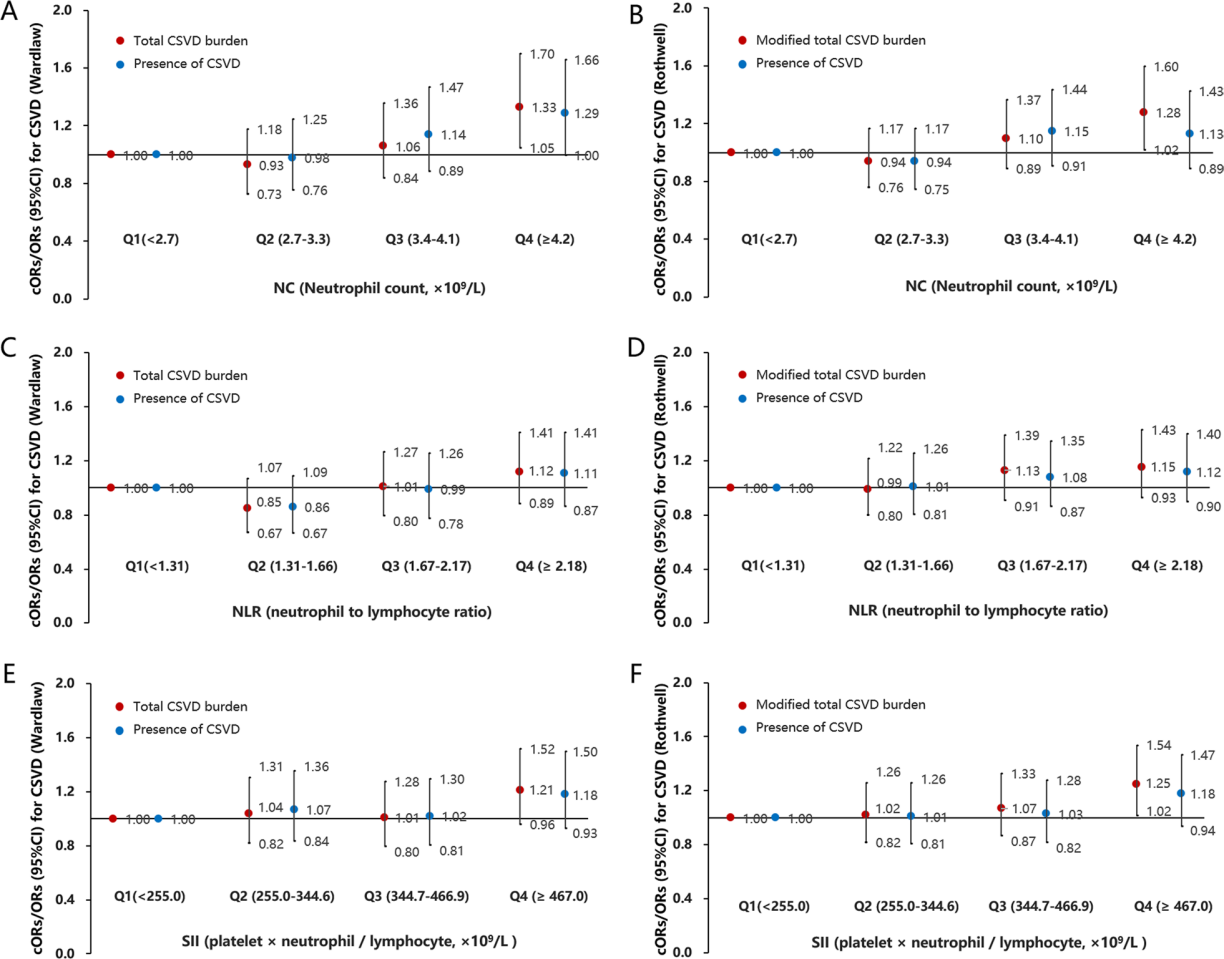
Forest plots for the association of NC, NLR and SII with CSVD. Forest plots show the cORs/ORs for NC and total CSVD burden or presence of CSVD (**A**, **B**); the cORs/ORs for NLR (neutrophil-to-lymphocyte ratio) and total CSVD burden or presence of CSVD (**C**, **D**); the cORs/ORs for SII (systemic immune-inflammation index) and total CSVD burden or presence of CSVD (**E**, **F**). Association for ordinal categorical outcome of total CSVD burden was expressed as cOR, whereas presence of CSVD was expressed as OR. The black lines represent the 95% confidence intervals of cORs/ORs. Multivariable logistic regression model adjusted for traditional risk factors of CSVD in Table 1. *NC* neutrophil count, *NLR* neutrophil-to-lymphocyte ratio, *SII* systemic immune-inflammation index (platelet count × neutrophil count/lymphocyte count), *cOR* common odds ratio, *OR* odds ratio. Wardlaw/total CSVD burden: 1 point allocated for presence of lacunes, microbleeds, moderate-to-severe (> 10) perivascular space in basal ganglia, periventricular white matter hyperintensities Fazekas 3 or deep white matter hyperintensities Fazekas 2-3. Rothwell/modified total CSVD burden: 1 point allocated for presence of lacunes, 1-4 microbleeds, frequent to severe (> 20) perivascular space in basal ganglia, moderate white matter hyperintensities (total periventricular + subcortical white matter hyperintensities grade 3-4), 2 points allocated for ≥ 5 microbleeds and severe white matter hyperintensities (total periventricular + subcortical white matter hyperintensities grade 5-6)

After adjustment for conventional covariates, subjects in the last quartiles (Q4) of NC were nominally associated with a higher total CSVD burden and a modified total CSVD burden than those in the first quartile (Q1) (cOR 1.33, 95% CI 1.05–1.70, *p* = 0.02, P_FDR = 0.06; cOR 1.28, 95% CI 1.02–1.60, *p* = 0.03, P_FDR = 0.06) (Fig. 1 and Additional file 1: Table S3). Q4 of NC was also suggestively associated with the presence of CSVD (Wardlaw, OR 1.29, 95% CI 1.00–1.66, *p* = 0.049, P_FDR = 0.07) (Fig. 1 and Additional file 1: Table S3). However, there were no statistically significant differences between NLR and CSVD burden or the presence of CSVD; or between SII and CSVD burden or the presence of CSVD (Fig. 1 and Additional file 1: Table S4 and S5).

**Fig. 2:**
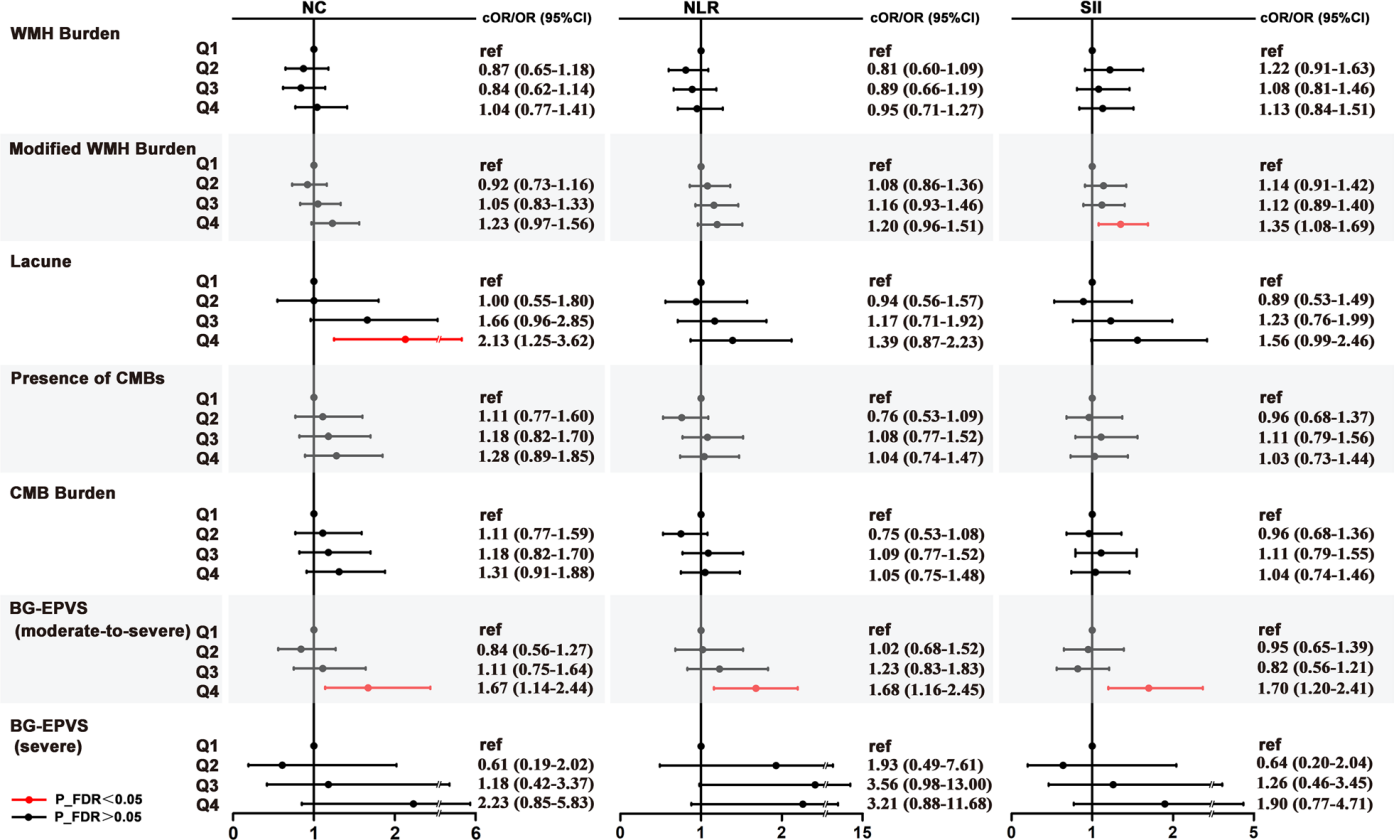
Forest plots for the association of NC, NLR and SII with CSVD imaging markers. Forest plots show the cORs/ORs for NC, NLR, SII and outcomes of WMH, Lacunes, CMBs and BG-EPVS assessed according to Wardlaw and Rothwell grading system, respectively. Association for ordinal categorical outcomes of Modified WMH burden and CMBs assessed according to Rothwell were expressed as cOR, whereas others was expressed as OR. The black lines represent the 95% confidence intervals of cORs/ORs. Multivariable logistic regression model adjusted for traditional risk factors of CSVD in Table 1. *NC* neutrophil count, *NLR* neutrophil-to-lymphocyte ratio, *SII* systemic immune-inflammation index (platelet count × neutrophil count/lymphocyte count), *cOR* common odds ratio, *OR* odds ratio. WMH burden was defined as either (early) confluent deep white matter hyperintensities (Fazekas score 2 or 3) or irregular periventricular white matter hyperintensities extending into the deep white matter (Fazekas score 3); modified WMH burden was classified into grade 0: total periventricular + subcortical white matter hyperintensities score 1–2, grade 1: total periventricular + subcortical white matter hyperintensities score 3–4 and grade 2: total periventricular + subcortical white matter hyperintensities score 5–6. Presence of CMBs was defined as presence of cerebral microbleeds; CMBs burden was classified as grade 0: absent, grade 1: 1–4 microbleeds and grade 2: ≥ 5 microbleeds. BG-EPVS (moderate-to-severe) indicated > 10 perivascular space in basal ganglia; BG-EPVS (severe) indicated severe (> 20) perivascular space in basal ganglia

Q4 of NC was associated with lacune (OR 2.13, 95% CI 1.25–3.62, *p* = 0.005, P_FDR = 0.03) and moderate-to-severe BG-EPVS (number of BG-EPVS > 10) (OR 1.67, 95% CI 1.14–2.44, *p* = 0.008, P_FDR = 0.03) compared with Q1 in Model 2 (Fig. 2 and Additional file 1: Table S6). Q4 of NLR was suggestively associated with moderate-to-severe BG-EPVS (number of BG-EPVS > 10) (OR 1.68, 95% CI 1.16–2.45, p = 0.007, P_FDR = 0.049) compared with Q1 in Model 2 (Fig. 2 and Additional file 1: Table S7). Q4 of SII was associated with modified WMH burden (OR 1.35, 95% CI 1.08–1.69, *p* = 0.008, P_FDR = 0.03) and moderate-to-severe BG-EPVS (number of BG-EPVS > 10) (OR 1.70, 95% CI 1.20–2.41, p = 0.003, P_FDR = 0.02) compared with Q1 in Model 2 (Fig. 2 and Additional file 1: Table S8). However, there were no significant differences between NC and WMH, CMB and severe BG-EPVS (number of BG-EPVS > 20); between NLR and WMH, lacune, CMB and severe BG-EPVS (number of BG-EPVS > 20); or between SII and WMH burden, lacune, CMB and severe BG-EPVS (number of BG-EPVS > 20) (Fig. 2 and Additional file 1: Table S6–S8).

### Two-sample MR analysis

**Fig. 3:**
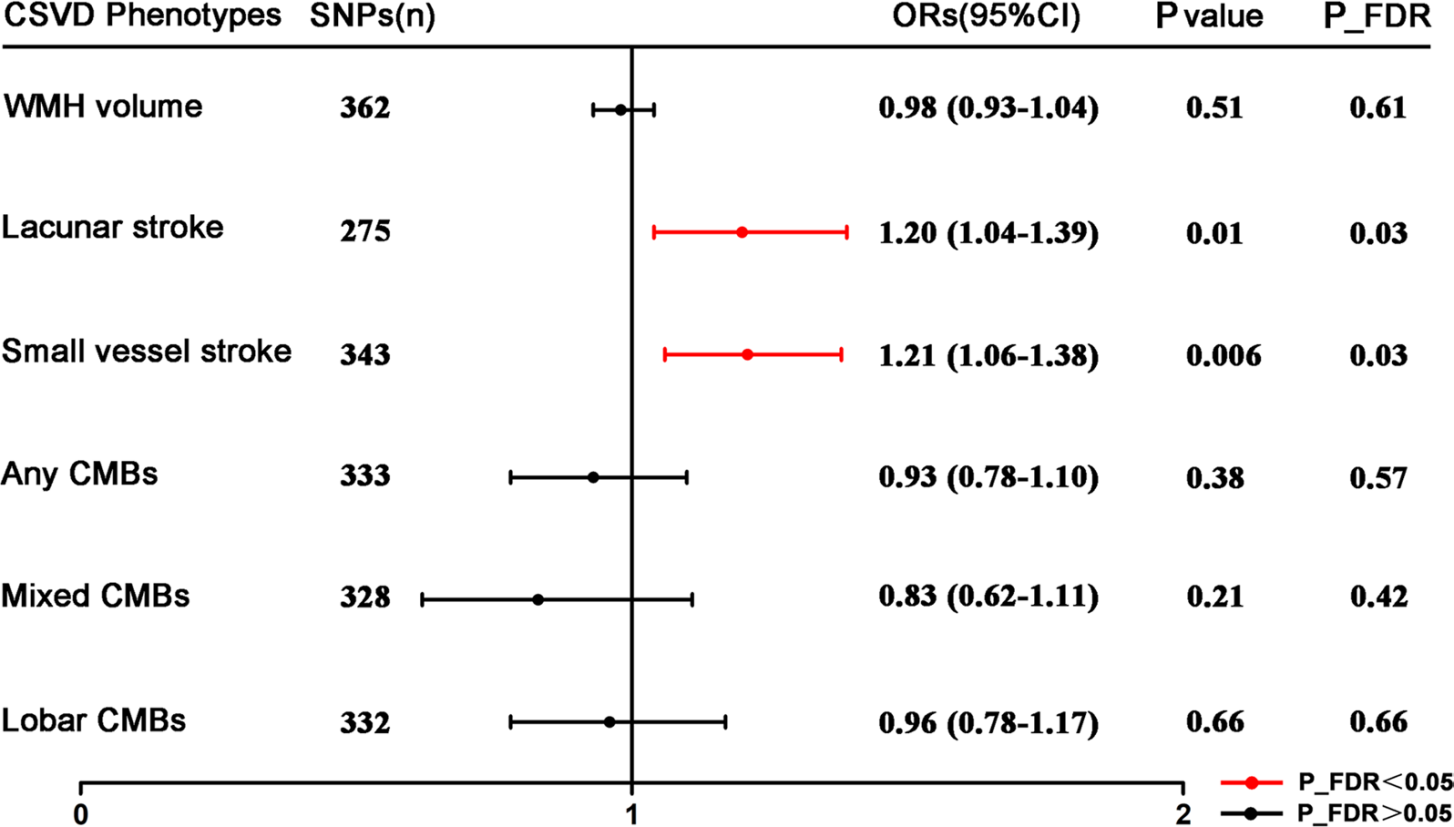
Mendelian randomization association of genetic determinants of neutrophil count with the risk of CSVD phenotypes. Forest plot shows estimates for the effects of neutrophil count related genetic variants on risk of cerebral small vessel disease phenotypes including white matter hyperintensities volume, lacunar stroke, small vessel stroke and cerebral microbleeds. The results are derived from the random-effects IVW (inverse-variance weighted) Mendelian randomization analysis and presented as odds ratios (95% confidence interval [CI]). Red rows correspond to statistically significant association estimates at a P_FDR (false discovery rate-adjusted p value) < 0.05. *CSVD* cerebral small vessel disease; *SNPs* single nucleotide polymorphisms, *ORs* odds ratios; *WMH* white matter hyperintensities, *CMBs* cerebral microbleeds

In the two-sample MR analyses, the random-effect IVW models showed that a genetically predicted 1-SD increase in NC level was associated with a higher risk of lacunar stroke (OR 1.20, 95% CI 1.04–1.39, p = 0.01, P_FDR = 0.03) and small vessel stroke (OR 1.21, 95% CI: 1.06–1.38, p = 0.006, P_FDR = 0.03) (Fig. 3). Cochran’ Q statistic indicated the heterogeneity of included genetic variants in IVW method, but MR-Egger analysis showed no pleiotropy for those instruments (Additional file 1: Table S9). The MR PRESSO and weighted median methods further showed close association between genetically predicted NC and lacunar stroke or small vessel stroke, consistent with IVW analysis (Additional file 1: Table S10). Furthermore, NC was not associated with WMH volume, any CMBs, mixed CMBs or lobar CMBs.

### Improvement in the prediction model for CSVD by the addition of NC, NLR and SII

We compared the performance of different models for predicting the presence of CSVD in community-based population (Table 2). The addition of NC to the basic model slightly improved the performance validated by NRI. The NRIs of NC for the presence of CSVD were 13.47% (5.78, 21.16%) (Wardlaw) and 13.07% (5.89, 20.25%) (Rothwell), respectively. A similar performance for predicting the presence of CSVD was verified for NC and the basic model. The IDIs of NC for the presence of CSVD (Wardlaw; Rothwell) were 0.22% (0.05, 0.39%) and 0.14% (0.01, 0.27%), respectively. The predictive performance of NLR evaluated by NRI and IDI was inconsistent. No significant improvement was observed after the addition of SII to the basic model by NRI and IDI.

**Table 2:** The NRI and IDI estimate of NC, NLR and SII

Variables	NRI		IDI	
	Estimate (95% CI), %	P value	Estimate (95% CI), %	P value
Presence of CSVD (Wardlaw)*				
Basic model	ref		ref	
Basic model + NC	13.47 (5.78, 21.16)	< 0.001	0.22 (0.05, 0.39)	0.01
Basic model + NLR	9.71 (2.02, 17.41)	0.01	0.05 (-0.03, 0.13)	0.19
Basic model + SII	5.19 (-2.51, 12.90)	0.19	0.06 (-0.02, 0.14)	0.14
Presence of CSVD (Rothwell)^†^				
Basic model	ref		ref	
Basic model + NC	13.07 (5.89, 20.25)	< 0.001	0.14 (0.01, 0.27)	0.03
Basic model + NLR	7.95 (0.76, 15.15)	0.03	0.04 (-0.03, 0.11)	0.22
Basic model + SII	6.81 (-0.39, 14.00)	0.06	0.06 (-0.02, 0.15)	0.14

*NC* neutrophil count, *NLR* neutrophil-to-lymphocyte ratio, *SII* systemic immune-inflammation index (platelet count × neutrophil count/lymphocyte count), *NRI* net reclassification index, *IDI* integrated discrimination improvement

*Wardlaw: 1 point allocated for presence of lacunes, microbleeds, moderate-to-severe (> 10) perivascular space in basal ganglia, periventricular white matter hyperintensities Fazekas 3 or deep white matter hyperintensities Fazekas 2–3

^†^Rothwell: 1 point allocated for presence of lacunes, 1–4 microbleeds, frequent to severe (> 20) perivascular space in basal ganglia, moderate white matter hyperintensities (total periventricular + subcortical white matter hyperintensities grade 3–4), 2 points allocated for ≥ 5 microbleeds and severe white matter hyperintensities (total periventricular + subcortical white matter hyperintensities grade 5–6)

Basic model: multivariable logistic regression model included age, sex, body mass index, diabetes, hypertension, total cholesterol, high-density lipoprotein, low-density lipoprotein, fasting blood glucose, homocysteine, previous dyslipidemia, previous heart disease, current smoking, current drinking, previous antiplatelet, anticoagulant, antihypertensive, antidiabetic, lipid-lowing drugs use

## Discussion

The present study investigated the association of NC, NLR and SII with the presence and severity of CSVD, and imaging markers in a community population. The main result indicated that elevated NC was suggestively associated with the presence and burden of CSVD, independent of traditional vascular risk factors. Furthermore, among the CSVD imaging markers, positive correlations were found between lacune and NC, between moderate-to-severe BG-EPVS and NC, NLR and SII; and between modified WMH burden and SII. In two-sample MR analyses, we found positive associations between genetic determinants of NC and lacunar stroke or small vessel stroke. The addition of NC to the basic model improved it slightly for the NRI and IDI, for predicting the presence of CSVD. Collectively, our findings highlight the clinical relevance of increasing NC with CSVD and imaging markers.

Neutrophils, lymphocytes, and platelets are essential elements of the immune system and important responders to injury and are implicated in the pathophysiology of vascular diseases [33–35]. The NLR is thought to be a useful predictor for assessing subclinical inflammation, while the SII is also an indicator of systemic immune states [8, 36]. Previous studies have revealed that higher NC, NLR and SII were independently associated with incidence, severity, and poor outcomes in stroke patients [6, 37–40]. We extended previous studies focusing on CSVD and its imaging markers. Our study indicated that the presence of CSVD increased along with levels of NC, NLR and SII in a community-based population, similarly to ischemic stroke [41]. Elevated inflammatory markers including NC and SII, were suggestively associated with the severity of CSVD, except NLR. Compared with past studies of stroke patients, the NLR level was lower in our community-based population, similar to a study in a healthy population [11, 38]. The lower NLR burden may show subclinical inflammation states and no significant correlation with CSVD, different from the higher NLR level with stroke. For CSVD imaging features, previous studies indicated that a higher NLR and SII were associated with white matter structure [11, 13]. Partially consistent with past results, the present study also revealed that elevated NC, NLR and SII were positively linked to some CSVD imaging features, especially for BG-EPVS, but not to CMBs. Moreover, two-sample MR analyses found an association of increased NC with a higher risk of lacunar stroke and small vessel stroke. Since lacunar stroke and lacune share similar pathological mechanisms, the results of MR analyses strengthened the evidence linking NC with lacunes and CSVD. The addition of inflammatory markers to the basic model including traditional risk factors for CSVD, improved the predictive performance of CSVD for the NRI and IDI, especially NC. The present study suggested that inflammatory markers may serve as an indicator to identify the risk for subclinical CSVD, especially for BG-EPVS and lacune.

The underlying mechanisms for the association of NC, NLR and SII with CSVD are not well-elucidated. There were several potential explanations. Previous studies revealed that alterations in neutrophils, lymphocytes and platelets were linked to damage to immune-regulatory homeostasis [42]. Chronic subclinical inflammation stimulates the adhesion of leukocytes to the vascular endothelium, and results in endothelial dysfunction [43]. Meanwhile, inflammatory cells infiltrate and accumulate in the perivascular space resulting in disruption of the blood–brain barrier and enlarged PVS [44]. In addition, inflammatory molecules such as matrix metalloproteinases, tumor necrosis factor-α and interleukin-1β are released, and microglia are activated, which jointly accelerate the inflammatory cascades and eventually result in white matter lesions [45]. The integrity changes to the blood vessel architecture may also lead to dilation and narrowing of the vessel lumen and stiffened vessels with abnormal autoregulation [46]. Moreover, chronic low-level inflammation is associated with various vascular risk factors for CSVD, including hypertension and diabetes [47]. The inflammatory response in CSVD is complex and needs further investigation.

Several limitations in our study need consideration. First, our study is a cross-sectional study, and any causal conclusions of NC, NLR and SII with CSVD and imaging markers cannot be drawn. Although we performed MR analysis to evaluate the causal relationship of NC with lacunar stroke, small vessel stroke, WMH volume and CMBs, the causal relationship of NC with PVS, NLR or SII with CSVD and imaging markers cannot be explored due to a lack of GWAS data on these phenotypes. The UK Biobank data may be helpful to perform the GWAS of the lacking phenotypes. Second, the reference data were derived from individuals of European ancestry for the MR analysis, which is inconsistent with the East Asian ethnicity in the PRECISE study. The differences in genetics background between European and East Asian ancestries may result in bias. Furthermore, the lacunar stroke in MR analysis does not fully represent lacune (the CSVD imaging marker), even though they share similar pathological mechanisms. In this cross-sectional study, stroke and its outcomes were not assessed, and further investigation are required in the prospective PRECISE study. Finally, the participants enrolled in this study were from only one city in China, which may have resulted in selection bias and inaccuracy to some degree. The prediction model was performed only in the discovery sample in this study, an external validation study is necessary to evaluate the predictive performance. Further large-scale prospective studies and GWASs of CSVD in East Asian ethnicities are necessary to confirm our findings.

## Conclusions

In conclusion, hematological inflammatory biomarker (NC) was suggestively associated with CSVD, especially moderate-to-severe BG-EPVS and lacune in community-based population. NC may be important factor for predicting the presence of CSVD. If these results can be validated in additional prospective studies, they may have clinical utility for identifying patients at high risk of CSVD.

## Supplementary Information

**Additional file 1.** The supplementary figures and tables of this article.

## Data Availability

The data that support the findings of this study are available from the corresponding author upon reasonable request.

## Abbreviations

NC: Neutrophil count
NLR: Neutrophil-to-lymphocyte ratio
SII: Systemic immune-inflammation index
CSVD: Cerebral small vessel disease
PRECISE: Poly-vasculaR Evaluation for Cognitive Impairment and vaScular Events
WMH: White matter hyperintensity
CMBs: Cerebral microbleeds
EPVS: Enlarged perivascular spaces
BG-EPVS: Enlarged perivascular spaces in basal ganglia
MR: Mendelian randomization
MRI: Magnetic resonance imaging
3D T1w MPRAGE: Three-dimensional T1-weighted magnetization prepared rapid acquisition gradient-echo
FLAIR: Axial T2-weighted, fluid-attenuated inversion recovery
DICOM: Digital imaging and communications in medicine
STRIVE: Standards for reporting vascular changes on neuroimaging
GWAS: Genome-wide association studies
NRI: Net reclassification index
IDI: Integrated discrimination improvement
IVW: Inverse-variance weighted
MR PRESSO: Mendelian Randomization Pleiotropy RESidual Sum and Outlier
