# Supplementary material for: Association of inflammatory markers with cerebral small vessel disease in community-based population
Source: J Neuroinflammation. 2022 May 6;19:106. doi: 10.1186/s12974-022-02468-0 (PMC9072153; doi:10.1186/s12974-022-02468-0)
Supplement: Supplementary file 1 — Additional file 1. The supplementary figures and tables of this article. [file 12974_2022_2468_MOESM1_ESM.docx]

**Additional file 1**

**FIGURE LEGENDS**


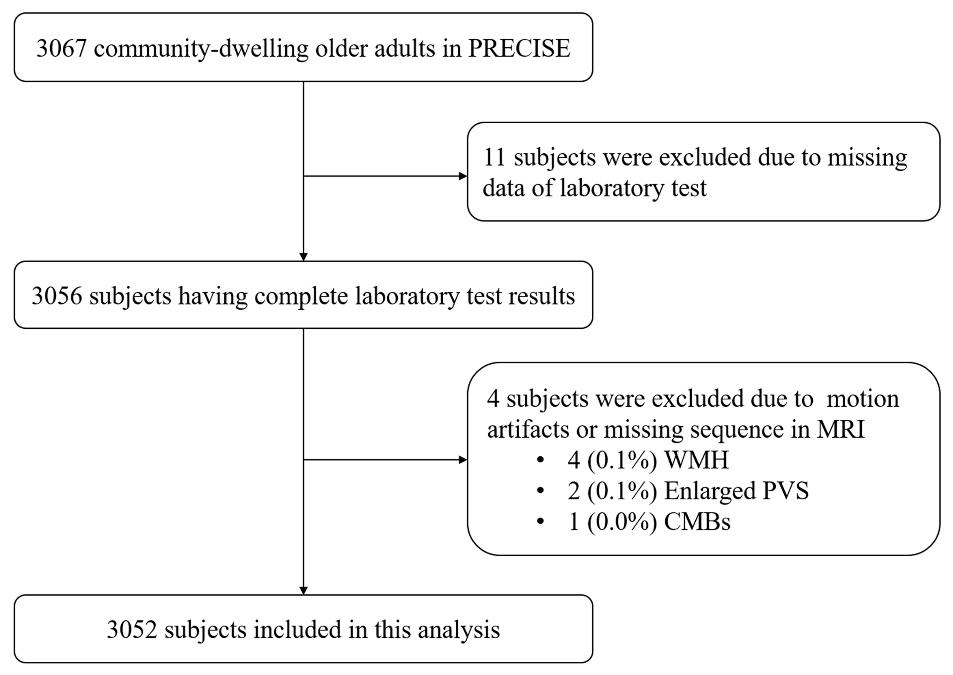
**Figure S1.** **The flowchart of the included subjects.** PRECISE, poly-vascular evaluation for cognitive impairment and vascular events; WMH, white matter hyperintensities; PVS, perivascular spaces; CMBs, cerebral microbleeds.

**Figure S2. Distribution of total CSVD burden by quartiles of NC, NLR and SII.** The percentage of total CSVD burden according to quartiles of NC (A), NLR (B) and SII (C). Grade 0, total CSVD burden (Wardlaw) and CSVD Burden (Rothwell) score 0; Grade 1, total CSVD burden (Wardlaw) and CSVD Burden (Rothwell) score 1; Grade 2, total CSVD burden (Wardlaw) score 2-4 and CSVD Burden (Rothwell) score 2-6. NC, neutrophil count; NLR, neutrophil-to-lymphocyte ratio; SII, systemic immune-inflammation index (platelet count × neutrophil count/lymphocyte count).

^*^Total CSVD burden (Wardlaw): 1 point allocated for presence of lacunes, microbleeds, moderate to severe (>10) perivascular space in basal ganglia, periventricular white matter hyperintensities Fazekas 3 or deep white matter hyperintensities Fazekas 2-3.

^†^Modified total CSVD Burden (Rothwell): 1 point allocated for presence of lacunes, 1-4 microbleeds, frequent to severe (>20) perivascular space in basal ganglia, moderate white matter hyperintensities (total periventricular + subcortical white matter hyperintensities grade 3-4), 2 points allocated for ≥5 microbleeds and severe white matter hyperintensities (total periventricular + subcortical white matter hyperintensities grade 5-6).


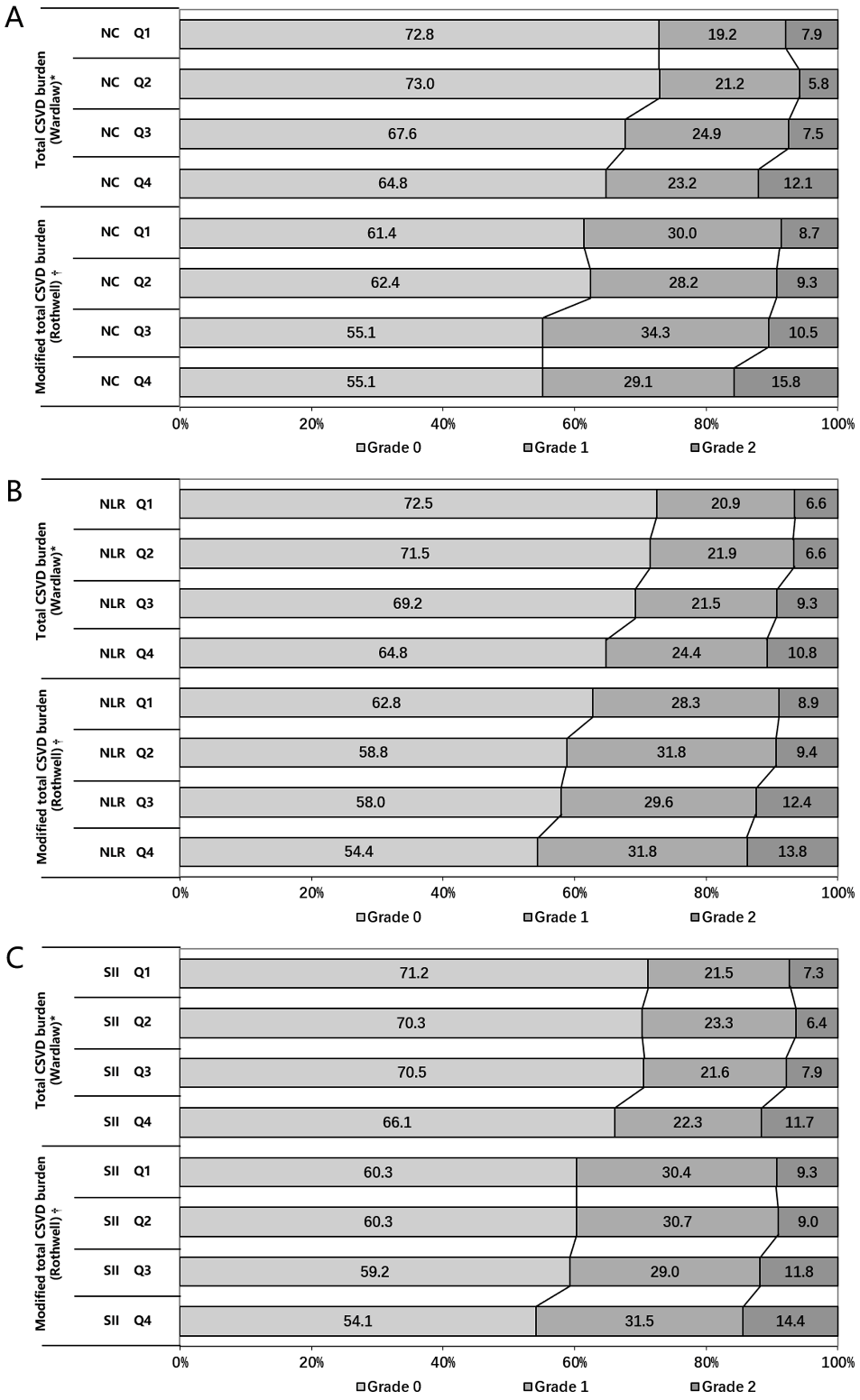


**TABLES**

**Table S1. The detailed MRI scan parameters in the present study.**

| **Contrast** | **Orientation** | **Voxel /mm^3^** | **Parameters** | **Scan time** |
| --- | --- | --- | --- | --- |
| T1w MPRAGE | 3D sagittal | 1.00×1.00×1.00 | TE=3.0ms, TR=6.7ms, TI=880ms, shot interval=2000ms, Flip angle=8° | 4:30 |
| SWI | 3D axial | 0.63×0.63×0.80 | first TE=7.2ms, echo spacing = 6.2ms, 5 echoes, Flip angle=17°, TR =37ms | 2:20 |
| FLAIR imaging | 2D axial | 0.53×0.53×6.50 | TE=110ms, TR=7000ms, TI=2300ms, SPIR fat suppression | 2:27 |
| T2w imaging | 2D axial | 0.51×0.51×6.50 | TE=105ms, TR=2500ms, SPIR fat suppression | 1:05 |

T1w MPRAGE, T1-weighted magnetization prepared rapid acquisition gradient-echo; SWI, susceptibility-weighted imaging; FLAIR, fluid-attenuated inversion recovery; T2w, T2-weighted.

**TABLE S2. Characteristics of the GWAS used in this study.**

| **Phenotype** | **Consortium** | **Sample size** | **Ancestry** | **Genotype data** | **PMID** |
| --- | --- | --- | --- | --- | --- |
| **Exposure** |  |  |  |  |  |
| Neutrophil count | UK Biobank | Up to 408,112 individuals | European | GWAS array | 32888494 |
| **Outcomes** |  |  |  |  |  |
| White matter hyperintensities | CHARGE + UK Biobank | Up to 50,970 individuals | European | GWAS array and metabochip array | 33293549 |
| Lacunar stroke | UK DNA Lacunar Stroke studies 1and 2 + ISGC | Up to 254,959 individuals | European | GWAS array and metabochip array | 33773637 |
| Small vessel stroke | MEGASTROKE | Up to 198,048 individuals | European | GWAS array and metabochip array | 29531354 |
| Cerebral microbleeds | CHARGE + UK Biobank | Up to 25,862 individuals | European | GWAS array and metabochip array | 32913026 |

GWAS, genome-wide association studies; CHARGE, Cohorts for Heart and Aging Research in Genomic Epidemiology; ISGC, International Stroke Genetics Consortium.

**Table S3. The association of NC with presence of CSVD and CSVD burden.**

| **Variables** | **Quartiles of**  **NC (×10^9^/L)** | **Model 1**^‡^ | | **Model 2**^§^ | | |
| --- | --- | --- | --- | --- | --- | --- |
|  |  | **Adjusted cOR/OR**  **(95% CI)** | **P value** | **Adjusted cOR/OR**  **(95% CI)** | **P value** | **P_FDR** |
| **Total CSVD burden (Wardlaw)^*^** | Q1 (<2.7) | ref |  | ref |  |  |
|  | Q2 (2.7-3.3) | 0.98 (0.77-1.24) ^#^ | 0.86 | 0.93 (0.73-1.18) ^#^ | 0.54 |  |
|  | Q3 (3.4-4.1) | 1.17 (0.92-1.48) ^#^ | 0.20 | 1.06 (0.84-1.36) ^#^ | 0.62 |  |
|  | Q4 (≥4.2) | 1.56 (1.24-1.97) ^#^ | <0.001 | 1.33 (1.05-1.70) ^#^ | 0.02 | 0.06 |
| **Presence of CSVD (Wardlaw)^*^** | Q1 (<2.7) | ref |  | ref |  |  |
|  | Q2 (2.7-3.3) | 1.02 (0.80-1.30) | 0.89 | 0.98 (0.76-1.25) | 0.85 |  |
|  | Q3 (3.4-4.1) | 1.23 (0.97-1.57) | 0.09 | 1.14 (0.89-1.47) | 0.29 |  |
|  | Q4 (≥4.2) | 1.47 (1.16-1.87) | 0.002 | 1.29 (1.00-1.66) | 0.049 | 0.07 |
| **Modified total CSVD burden (Rothwell)^†^** | Q1 (<2.7) | ref |  | ref |  |  |
|  | Q2 (2.7-3.3) | 1.00 (0.81-1.23) ^#^ | 0.98 | 0.94 (0.76-1.17) ^#^ | 0.56 |  |
|  | Q3 (3.4-4.1) | 1.23 (1.00-1.53) ^#^ | 0.055 | 1.10 (0.89-1.37) ^#^ | 0.38 |  |
|  | Q4 (≥4.2) | 1.51 (1.22-1.87) ^#^ | <0.001 | 1.28 (1.02-1.60) ^#^ | 0.03 | 0.06 |
| **Presence of CSVD (Rothwell)^†^** | Q1 (<2.7) | ref |  | ref |  |  |
|  | Q2 (2.7-3.3) | 0.99 (0.79-1.23) | 0.91 | 0.94 (0.75-1.17) | 0.56 |  |
|  | Q3 (3.4-4.1) | 1.28 (1.03-1.60) | 0.03 | 1.15 (0.91-1.44) | 0.25 |  |
|  | Q4 (≥4.2) | 1.35 (1.08-1.68) | 0.009 | 1.13 (0.89-1.43) | 0.30 | 0.30 |

NC, neutrophil count; cOR, common odds ratio; OR, odds ratio.

^*^ Wardlaw: 1 point allocated for presence of lacunes, microbleeds, moderate to severe (>10) perivascular space in basal ganglia, periventricular white matter hyperintensities Fazekas 3 or deep white matter hyperintensities Fazekas 2-3.

^†^Rothwell: 1 point allocated for presence of lacunes, 1-4 microbleeds, frequent to severe (>20) perivascular space in basal ganglia, moderate white matter hyperintensities (total periventricular + subcortical white matter hyperintensities grade 3-4), 2 points allocated for ≥5 microbleeds and severe white matter hyperintensities (total periventricular + subcortical white matter hyperintensities grade 5-6).

^‡^ Model1: multivariable logistic regression model adjusted for covariates: age, sex.

^§^ Model2: multivariable logistic regression model adjusted for covariates: age, sex, body mass index, diabetes, hypertension, total cholesterol, high-density lipoprotein, low density lipoprotein, fasting blood glucose, homocysteine, previous dyslipidemia, previous heart disease, current smoking, current drinking, previous antiplatelet, anticoagulant, antihypertensive, antidiabetic, lipid-lowing drugs use.

^#^ As total CSVD burden and modified total CSVD burden were ordinal categorical variables, ordinal logistic regression model was used and cORs were presented, whereas logistic regression analysis was used and ORs were presented for presence of CSVD.

**TABLE S4. The association of NLR with presence of CSVD and CSVD burden.**

| **Variables** | **Quartiles of**  **NLR** | **Model 1**^‡^ | | **Model 2**^§^ | | |
| --- | --- | --- | --- | --- | --- | --- |
|  |  | **Adjusted cOR/OR**  **(95% CI)** | **P value** | **Adjusted cOR/OR**  **(95% CI)** | **P value** | **P_FDR** |
| **Total CSVD burden (Wardlaw)^*^** | Q1 (<1.31) | ref |  | ref |  |  |
|  | Q2 (1.31-1.66) | 0.93 (0.73-1.17) ^#^ | 0.51 | 0.85 (0.67-1.07) ^#^ | 0.16 |  |
|  | Q3 (1.67-2.17) | 1.10 (0.87-1.38) ^#^ | 0.42 | 1.01 (0.80-1.27) ^#^ | 0.94 |  |
|  | Q4 (≥2.18) | 1.25 (1.00-1.57) ^#^ | 0.055 | 1.12 (0.89-1.41) ^#^ | 0.34 | 0.41 |
| **Presence of CSVD (Wardlaw)^*^** | Q1 (<1.31) | ref |  | ref |  |  |
|  | Q2 (1.31-1.66) | 0.93 (0.74-1.18) | 0.57 | 0.86 (0.67-1.09) | 0.21 |  |
|  | Q3 (1.67-2.17) | 1.07 (0.85-1.35) | 0.58 | 0.99 (0.78-1.26) | 0.92 |  |
|  | Q4 (≥2.18) | 1.21 (0.96-1.53) | 0.10 | 1.11 (0.87-1.41) | 0.41 | 0.41 |
| **Modified total CSVD burden (Rothwell)^†^** | Q1 (<1.31) | ref |  | ref |  |  |
|  | Q2 (1.31-1.66) | 1.07 (0.87-1.32) ^#^ | 0.50 | 0.99 (0.80-1.22) ^#^ | 0.92 |  |
|  | Q3 (1.67-2.17) | 1.21 (0.99-1.49) ^#^ | 0.07 | 1.13 (0.91-1.39) ^#^ | 0.27 |  |
|  | Q4 (≥2.18) | 1.31 (1.06-1.61) ^#^ | 0.01 | 1.15 (0.93-1.43) ^#^ | 0.19 | 0.41 |
| **Presence of CSVD (Rothwell) ^†^** | Q1 (<1.31) | ref |  | ref |  |  |
|  | Q2 (1.31-1.66) | 1.09 (0.88-1.35) | 0.44 | 1.01 (0.81-1.26) | 0.93 |  |
|  | Q3 (1.67-2.17) | 1.16 (0.94-1.44) | 0.17 | 1.08 (0.87-1.35) | 0.48 |  |
|  | Q4 (≥2.18) | 1.27 (1.02-1.58) | 0.03 | 1.12 (0.90-1.40) | 0.32 | 0.41 |

NLR, neutrophil-to-lymphocyte ratio; cOR, common odds ratio; OR, odds ratio.

^*^ Wardlaw: 1 point allocated for presence of lacunes, microbleeds, moderate to severe (>10) perivascular space in basal ganglia, periventricular white matter hyperintensities Fazekas 3 or deep white matter hyperintensities Fazekas 2-3.

^†^Rothwell:1 point allocated for presence of lacunes, 1-4 microbleeds, frequent to severe (>20) perivascular space in basal ganglia, moderate white matter hyperintensities (total periventricular + subcortical white matter hyperintensities grade 3-4), 2 points allocated for ≥5 microbleeds and severe white matter hyperintensities (total periventricular + subcortical white matter hyperintensities grade 5-6).

^‡^ Model1: multivariable logistic regression model adjusted for covariates: age, sex.

^§^ Model2: multivariable logistic regression model adjusted for covariates: age, sex, body mass index, diabetes, hypertension, total cholesterol, high-density lipoprotein, low density lipoprotein, fasting blood glucose, homocysteine, previous dyslipidemia, previous heart disease, current smoking, current drinking, previous antiplatelet, anticoagulant, antihypertensive, antidiabetic, lipid-lowing drugs use.

^#^ As total CSVD burden and modified total CSVD burden were ordinal categorical variables, ordinal logistic regression model was used and cORs were presented, whereas logistic regression analysis was used and ORs were presented for presence of CSVD.

**TABLE S5.** **The association of SII with presence of CSVD and CSVD burden.**

| **Variables** | **Quartiles of**  **SII (×10^9^/L)** | **Model 1**^‡^ | | **Model 2**^§^ | | |
| --- | --- | --- | --- | --- | --- | --- |
|  |  | **Adjusted cOR/OR**  **(95% CI)** | **P value** | **Adjusted cOR/OR**  **(95% CI)** | **P value** | **P_FDR** |
| **Total CSVD burden (Wardlaw)^*^** | Q1 (<255.0) | ref |  | ref |  |  |
|  | Q2 (255.0-344.6) | 1.08 (0.86-1.36) ^#^ | 0.50 | 1.04 (0.82-1.31) ^#^ | 0.75 |  |
|  | Q3 (344.7-466.9) | 1.07 (0.85-1.34) ^#^ | 0.57 | 1.01 (0.80-1.28) ^#^ | 0.92 |  |
|  | Q4 (≥467.0) | 1.35 (1.08-1.69) ^#^ | 0.008 | 1.21 (0.96-1.52) ^#^ | 0.11 | 0.17 |
| **Presence of CSVD (Wardlaw)^*^** | Q1 (<255.0) | ref |  | ref |  |  |
|  | Q2 (255.0-344.6) | 1.10 (0.87-1.38) | 0.45 | 1.07 (0.84-1.36) | 0.57 |  |
|  | Q3 (344.7-466.9) | 1.06 (0.84-1.33) | 0.65 | 1.02 (0.81-1.30) | 0.85 |  |
|  | Q4 (≥467.0) | 1.28 (1.02-1.61) | 0.04 | 1.18 (0.93-1.50) | 0.17 | 0.17 |
| **Modified total CSVD burden (Rothwell)^†^** | Q1 (<255.0) | ref |  | ref |  |  |
|  | Q2 (255.0-344.6) | 1.04 (0.84-1.28) ^#^ | 0.74 | 1.02 (0.82-1.26) ^#^ | 0.88 |  |
|  | Q3 (344.7-466.9) | 1.13 (0.91-1.38) ^#^ | 0.27 | 1.07 (0.87-1.33) ^#^ | 0.51 |  |
|  | Q4 (≥467.0) | 1.41 (1.15-1.73) ^#^ | 0.001 | 1.25 (1.02-1.54) ^#^ | 0.04 | 0.16 |
| **Presence of CSVD (Rothwell) ^†^** | Q1 (<255.0) | ref |  | ref |  |  |
|  | Q2 (255.0-344.6) | 1.03 (0.83-1.28) | 0.81 | 1.01 (0.81-1.26) | 0.92 |  |
|  | Q3 (344.7-466.9) | 1.06 (0.86-1.32) | 0.58 | 1.03 (0.82-1.28) | 0.83 |  |
|  | Q4 (≥467.0) | 1.31 (1.06-1.63) | 0.01 | 1.18 (0.94-1.47) | 0.15 | 0.17 |

SII, systemic immune-inflammation index (platelet count×neutrophil count/lymphocyte count); cOR, common odds ratio; OR, odds ratio.

^*^Wardlaw: 1 point allocated for presence of lacunes, microbleeds, moderate to severe (>10) perivascular space in basal ganglia, periventricular white matter hyperintensities Fazekas 3 or deep white matter hyperintensities Fazekas 2-3.

^†^Rothwell: 1 point allocated for presence of lacunes, 1-4 microbleeds, frequent to severe (>20) perivascular space in basal ganglia, moderate white matter hyperintensities (total periventricular + subcortical white matter hyperintensities grade 3-4), 2 points allocated for ≥5 microbleeds and severe white matter hyperintensities (total periventricular + subcortical white matter hyperintensities grade 5-6).

^‡^ Model1: multivariable logistic regression model adjusted for covariates: age, sex.

^§^ Model2: multivariable logistic regression model adjusted for covariates: age, sex, body mass index, diabetes, hypertension, total cholesterol, high-density lipoprotein, low density lipoprotein, fasting blood glucose, homocysteine, previous dyslipidemia, previous heart disease, current smoking, current drinking, previous antiplatelet, anticoagulant, antihypertensive, antidiabetic, lipid-lowing drugs use.

^#^ As total CSVD burden and modified total CSVD burden were ordinal categorical variables, ordinal logistic regression model was used and cORs were presented, whereas logistic regression analysis was used and ORs were presented for presence of CSVD.

**TABLE S6. The association of NC with MRI-defined characteristic of CSVD.**

| **Variables** | **Quartiles of**  **NC (×10^9^/L)** | **N** | **Event Rate**  **n (%)** | **Model 1**^‡^ | | **Model 2**^§^ | | |
| --- | --- | --- | --- | --- | --- | --- | --- | --- |
|  |  |  |  | **Adjusted cOR/OR**  **(95% CI)** | **P value** | **Adjusted cOR/OR**  **(95% CI)** | **P value** | **P_FDR** |
| **WMH Burden*** | Q1 (<2.7) | 681 | 110 (16.2) | ref |  | ref |  |  |
|  | Q2 (2.7-3.3) | 825 | 122 (14.8) | 0.93 (0.69-1.24) | 0.62 | 0.87 (0.65-1.18) | 0.38 |  |
|  | Q3 (3.4-4.1) | 760 | 122 (16.1) | 0.94 (0.70-1.27) | 0.69 | 0.84 (0.62-1.14) | 0.25 |  |
|  | Q4 (≥4.2) | 786 | 154 (19.6) | 1.29 (0.97-1.72) | 0.08 | 1.04 (0.77-1.41) | 0.81 | 0.81 |
| **Modified WMH Burden ^*^** | Q1 (<2.7) | 681 |  | ref |  | ref |  |  |
|  | Q2 (2.7-3.3) | 825 |  | 0.99 (0.79-1.25) ^#^ | 0.95 | 0.92 (0.73-1.16) ^#^ | 0.50 |  |
|  | Q3 (3.4-4.1) | 760 |  | 1.20 (0.96-1.51) ^#^ | 0.11 | 1.05 (0.83-1.33) ^#^ | 0.69 |  |
|  | Q4 (≥4.2) | 786 |  | 1.51 (1.20-1.89) ^#^ | <0.001 | 1.23 (0.97-1.56) ^#^ | 0.09 | 0.18 |
| **Lacune** | Q1 (<2.7) | 681 | 21 (3.1) | ref |  | ref |  |  |
|  | Q2 (2.7-3.3) | 825 | 29 (3.5) | 1.16 (0.65-2.06) | 0.62 | 1.00 (0.55-1.80) | 0.99 |  |
|  | Q3 (3.4-4.1) | 760 | 49 (6.5) | 2.03 (1.20-3.45) | 0.009 | 1.66 (0.96-2.85) | 0.07 |  |
|  | Q4 (≥4.2) | 786 | 71 (9.3) | 2.96 (1.78-4.91) | <0.001 | 2.13 (1.25-3.62) | 0.005 | **0.03** |
| **Presence of CMBs^†^** | Q1 (<2.7) | 681 | 59 (8.7) | ref |  | ref |  |  |
|  | Q2 (2.7-3.3) | 825 | 80 (9.7) | 1.13 (0.79-1.61) | 0.50 | 1.11 (0.77-1.60) | 0.57 |  |
|  | Q3 (3.4-4.1) | 760 | 80 (10.5) | 1.17 (0.82-1.68) | 0.38 | 1.18 (0.82-1.70) | 0.38 |  |
|  | Q4 (≥4.2) | 786 | 93 (11.8) | 1.33 (0.94-1.89) | 0.11 | 1.28 (0.89-1.85) | 0.19 | 0.22 |
| **CMB Burden^†^** | Q1 (<2.7) | 681 |  | ref |  | ref |  |  |
|  | Q2 (2.7-3.3) | 825 |  | 1.13 (0.79-1.62) ^#^ | 0.50 | 1.11 (0.77-1.59) ^#^ | 0.57 |  |
|  | Q3 (3.4-4.1) | 760 |  | 1.17 (0.82-1.68) ^#^ | 0.38 | 1.18 (0.82-1.70) ^#^ | 0.38 |  |
|  | Q4 (≥4.2) | 786 |  | 1.36 (0.96-1.92) ^#^ | 0.09 | 1.31 (0.91-1.88) ^#^ | 0.15 | 0.21 |
| **BG-EPVS (moderate-to-severe)** ^††^ | Q1 (<2.7) | 681 | 55 (8.1) | ref |  | ref |  |  |
|  | Q2 (2.7-3.3) | 825 | 57 (6.9) | 0.85 (0.57-1.28) | 0.44 | 0.84 (0.56-1.27) | 0.41 |  |
|  | Q3 (3.4-4.1) | 760 | 76 (10.0) | 1.17 (0.80-1.71) | 0.42 | 1.11 (0.75-1.64) | 0.61 |  |
|  | Q4 (≥4.2) | 786 | 111 (14.1) | 1.84 (1.28-2.64) | <0.001 | 1.67 (1.14-2.44) | 0.008 | **0.03** |
| **BG-EPVS (severe)** ^††^ | Q1 (<2.7) | 681 | 7 (1.0) | ref |  | ref |  |  |
|  | Q2 (2.7-3.3) | 825 | 5 (0.6) | 0.61 (0.19-1.96) | 0.40 | 0.61 (0.19-2.02) | 0.42 |  |
|  | Q3 (3.4-4.1) | 760 | 9 (1.2) | 1.02 (0.37-2.80) | 0.98 | 1.18 (0.42-3.37) | 0.75 |  |
|  | Q4 (≥4.2) | 786 | 17 (2.2) | 1.93 (0.77-4.79) | 0.16 | 2.23 (0.85-5.83) | 0.10 | 0.18 |

NC, neutrophil count; WHM, white matter hyperintensities; CMBs, cerebral microbleeds; BG-PVS, perivascular spaces in basal ganglia; cOR, common odds ratio; OR, odds ratio.

^*^ WMH Burden was defined as either (early) confluent deep white matter hyperintensities (Fazekas score 2 or 3) or irregular periventricular white matter hyperintensities extending into the deep white matter (Fazekas score 3); Modified WMH Burden was classified into grade 0: total periventricular + subcortical white matter hyperintensities score 1-2, grade 1: total periventricular + subcortical white matter hyperintensities score 3-4 and grade 2: total periventricular + subcortical white matter hyperintensities score 5-6.

^†^ Presence of CMBs was defined as presence of cerebral microbleeds; CMBs Burden was classified as grade 0: absent, grade 1: 1-4 microbleeds and grade 2: ≥5 microbleeds.

^††^ BG-EPVS (moderate-to-severe) indicated >10 perivascular space in basal ganglia; BG-EPVS (severe) indicated severe (>20) perivascular space in basal ganglia.

^‡^ Model1: multivariable logistic regression model adjusted for covariates: age, sex.

^§^ Model2: multivariable logistic regression model adjusted for covariates: age, sex, body mass index, diabetes, hypertension, total cholesterol, high-density lipoprotein, low density lipoprotein, fasting blood glucose, homocysteine, previous dyslipidemia, previous heart disease, current smoking, current drinking, previous antiplatelet, anticoagulant, antihypertensive, antidiabetic, lipid-lowing drugs use.

^#^ As Modified WMH Burden and CMBs Burden were ordinal categorical variables, ordinal logistic regression model was used and cORs were presented, whereas logistic regression analysis was used and ORs were presented for others.

**TABLE S7. The association of NLR with MRI-defined characteristic of CSVD.**

| **Variables** | **Quartiles of**  **NLR** | **N** | **Event Rate**  **n (%)** | **Model 1**^‡^ | | **Model 2**^§^ | | |
| --- | --- | --- | --- | --- | --- | --- | --- | --- |
|  |  |  |  | **Adjusted cOR/OR**  **(95% CI)** | **P value** | **Adjusted cOR/OR**  **(95% CI)** | **P value** | **P_FDR** |
| **WMH Burden*** | Q1 (<1.31) | 760 | 117 (15.4) | ref |  | ref |  |  |
|  | Q2 (1.31-1.66) | 767 | 121 (15.8) | 0.91 (0.68-1.21) | 0.51 | 0.81 (0.60-1.09) | 0.17 |  |
|  | Q3 (1.67-2.17) | 764 | 126 (16.5) | 0.99 (0.75-1.33) | 0.97 | 0.89 (0.66-1.19) | 0.43 |  |
|  | Q4 (≥2.18) | 761 | 144 (18.9) | 1.09 (0.82-1.45) | 0.54 | 0.95 (0.71-1.27) | 0.72 | 0.82 |
| **Modified WMH Burden ^*^** | Q1 (<1.31) | 760 |  | ref |  | ref |  |  |
|  | Q2 (1.31-1.66) | 767 |  | 1.18 (0.95-1.48) ^#^ | 0.14 | 1.08 (0.86-1.36) ^#^ | 0.50 |  |
|  | Q3 (1.67-2.17) | 764 |  | 1.27 (1.01-1.58) ^#^ | 0.04 | 1.16 (0.93-1.46) ^#^ | 0.20 |  |
|  | Q4 (≥2.18) | 761 |  | 1.39 (1.11-1.73) ^#^ | 0.004 | 1.20 (0.96-1.51) ^#^ | 0.11 | 0.26 |
| **Lacune** | Q1 (<1.31) | 760 | 30 (4.0) | ref |  | ref |  |  |
|  | Q2 (1.31-1.66) | 767 | 36 (4.7) | 1.06 (0.64-1.76) | 0.81 | 0.94 (0.56-1.57) | 0.82 |  |
|  | Q3 (1.67-2.17) | 764 | 44 (5.8) | 1.32 (0.81-2.13) | 0.27 | 1.17 (0.71-1.92) | 0.53 |  |
|  | Q4 (≥2.18) | 761 | 60 (7.9) | 1.68 (1.06-2.66) | 0.03 | 1.39 (0.87-2.23) | 0.17 | 0.30 |
| **Presence of CMBs^†^** | Q1 (<1.31) | 760 | 72 (9.5) | ref |  | ref |  |  |
|  | Q2 (1.31-1.66) | 767 | 63 (8.2) | 0.79 (0.55-1.13) | 0.20 | 0.76 (0.53-1.09) | 0.13 |  |
|  | Q3 (1.67-2.17) | 764 | 86 (11.3) | 1.12 (0.80-1.57) | 0.50 | 1.08 (0.77-1.52) | 0.65 |  |
|  | Q4 (≥2.18) | 761 | 91 (12.0) | 1.12 (0.81-1.57) | 0.49 | 1.04 (0.74-1.47) | 0.82 | 0.82 |
| **CMB Burden^†^** | Q1 (<1.31) | 760 |  | ref |  | ref |  |  |
|  | Q2 (1.31-1.66) | 767 |  | 0.79 (0.55-1.13) ^#^ | 0.20 | 0.75 (0.53-1.08) ^#^ | 0.13 |  |
|  | Q3 (1.67-2.17) | 764 |  | 1.13 (0.81-1.57) ^#^ | 0.49 | 1.09 (0.77-1.52) ^#^ | 0.63 |  |
|  | Q4 (≥2.18) | 761 |  | 1.14 (0.81-1.58) ^#^ | 0.45 | 1.05 (0.75-1.48) ^#^ | 0.77 | 0.82 |
| **BG-EPVS (moderate-to-severe)** ^††^ | Q1 (<1.31) | 760 | 51 (6.7) | ref |  | ref |  |  |
|  | Q2 (1.31-1.66) | 767 | 66 (8.6) | 1.11 (0.75-1.66) | 0.59 | 1.02 (0.68-1.52) | 0.94 |  |
|  | Q3 (1.67-2.17) | 764 | 75 (9.8) | 1.32 (0.90-1.94) | 0.16 | 1.23 (0.83-1.83) | 0.30 |  |
|  | Q4 (≥2.18) | 761 | 107 (14.1) | 1.81 (1.25-2.62) | 0.002 | 1.68 (1.16-2.45) | 0.007 | **0.049** |
| **BG-EPVS (severe)** ^††^ | Q1 (<1.31) | 760 | 3 (0.4) | ref |  | ref |  |  |
|  | Q2 (1.31-1.66) | 767 | 8 (1.0) | 2.02 (0.53-7.78) | 0.31 | 1.93 (0.49-7.61) | 0.35 |  |
|  | Q3 (1.67-2.17) | 764 | 13 (1.7) | 3.40 (0.95-12.17) | 0.06 | 3.56 (0.98-13.00) | 0.055 |  |
|  | Q4 (≥2.18) | 761 | 14 (1.8) | 2.97 (0.84-10.58) | 0.09 | 3.21 (0.88-11.68) | 0.08 | 0.26 |

NLR, neutrophil-to-lymphocyte ratio; WHM, white matter hyperintensities; CMBs, cerebral microbleeds; BG-PVS, perivascular spaces in basal ganglia; cOR, common odds ratio; OR, odds ratio.

^*^ WMH Burden was defined as either (early) confluent deep white matter hyperintensities (Fazekas score 2 or 3) or irregular periventricular white matter hyperintensities extending into the deep white matter (Fazekas score 3); Modified WMH Burden was classified into grade 0: total periventricular + subcortical white matter hyperintensities score 1-2, grade 1: total periventricular + subcortical white matter hyperintensities score 3-4 and grade 2: total periventricular + subcortical white matter hyperintensities score 5-6.

^†^ Presence of CMBs was defined as presence of cerebral microbleeds; CMBs Burden was classified as grade 0: absent, grade 1: 1-4 microbleeds and grade 2: ≥5 microbleeds.

^††^ BG-EPVS (moderate-to-severe) indicated >10 perivascular space in basal ganglia; BG-EPVS (severe) indicated severe (>20) perivascular space in basal ganglia.

^‡^ Model1: multivariable logistic regression model adjusted for covariates: age, sex.

^§^ Model2: multivariable logistic regression model adjusted for covariates: age, sex, body mass index, diabetes, hypertension, total cholesterol, high-density lipoprotein, low density lipoprotein, fasting blood glucose, homocysteine, previous dyslipidemia, previous heart disease, current smoking, current drinking, previous antiplatelet, anticoagulant, antihypertensive, antidiabetic, lipid-lowing drugs use.

^#^ As Modified WMH Burden and CMBs Burden were ordinal categorical variables, ordinal logistic regression model was used and cORs were presented, whereas logistic regression analysis was used and ORs were presented for others.

**TABLE S8. The association of SII with MRI-defined characteristic of CSVD.**

| **Variables** | **Quartiles of**  **SII (×10^9^/L)** | **N** | **Event Rate**  **n (%)** | **Model 1**^‡^ | | **Model 2**^§^ | | |
| --- | --- | --- | --- | --- | --- | --- | --- | --- |
|  |  |  |  | **Adjusted cOR/OR**  **(95% CI)** | **P value** | **Adjusted cOR/OR**  **(95% CI)** | **P value** | **P_FDR** |
| **WMH Burden*** | Q1 (<255.0) | 763 | 113 (14.8) | ref |  | ref |  |  |
|  | Q2 (255.0-344.6) | 763 | 133 (17.) | 1.28 (0.96-1.71) | 0.09 | 1.22 (0.91-1.63) | 0.18 |  |
|  | Q3 (344.7-466.9) | 763 | 124 (16.3) | 1.15 (0.86-1.53) | 0.35 | 1.08 (0.81-1.46) | 0.60 |  |
|  | Q4 (≥467.0) | 763 | 138 (18.1) | 1.28 (0.97-1.71) | 0.09 | 1.13 (0.84-1.51) | 0.42 | 0.59 |
| **Modified WMH Burden ^*^** | Q1 (<255.0) | 763 |  | ref |  | ref |  |  |
|  | Q2 (255.0-344.6) | 763 |  | 1.17 (0.94-1.46) ^#^ | 0.17 | 1.14 (0.91-1.42) ^#^ | 0.27 |  |
|  | Q3 (344.7-466.9) | 763 |  | 1.18 (0.95-1.47) ^#^ | 0.15 | 1.12 (0.89-1.40) ^#^ | 0.34 |  |
|  | Q4 (≥467.0) | 763 |  | 1.54 (1.24-1.91) ^#^ | <0.001 | 1.35 (1.08-1.69) ^#^ | 0.008 | **0.03** |
| **Lacune** | Q1 (<255.0) | 763 | 33 (4.3) | ref |  | ref |  |  |
|  | Q2 (255.0-344.6) | 763 | 30 (3.9) | 0.95 (0.57-1.59) | 0.85 | 0.89 (0.53-1.49) | 0.65 |  |
|  | Q3 (344.7-466.9) | 763 | 44 (5.8) | 1.40 (0.88-2.24) | 0.16 | 1.23 (0.76-1.99) | 0.40 |  |
|  | Q4 (≥467.0) | 763 | 63 (8.3) | 1.97 (1.27-3.06) | 0.003 | 1.56 (0.99-2.46) | 0.056 | 0.13 |
| **Presence of CMBs^†^** | Q1 (<255.0) | 763 | 75 (9.8) | ref |  | ref |  |  |
|  | Q2 (255.0-344.6) | 763 | 71 (9.3) | 0.97 (0.69-1.37) | 0.86 | 0.96 (0.68-1.37) | 0.84 |  |
|  | Q3 (344.7-466.9) | 763 | 82 (10.8) | 1.12 (0.80-1.57) | 0.50 | 1.11 (0.79-1.56) | 0.54 |  |
|  | Q4 (≥467.0) | 763 | 84 (11.0) | 1.11 (0.80-1.55) | 0.53 | 1.03 (0.73-1.44) | 0.89 | 0.89 |
| **CMB Burden^†^** | Q1 (<255.0) | 763 |  | ref |  | ref |  |  |
|  | Q2 (255.0-344.6) | 763 |  | 0.96 (0.68-1.36) | 0.83 | 0.96 (0.68-1.36) | 0.81 |  |
|  | Q3 (344.7-466.9) | 763 |  | 1.12 (0.80-1.56) | 0.51 | 1.11 (0.79-1.55) | 0.56 |  |
|  | Q4 (≥467.0) | 763 |  | 1.13 (0.81-1.57) | 0.48 | 1.04 (0.74-1.46) | 0.82 | 0.89 |
| **BG-EPVS (moderate-to-severe)** ^††^ | Q1 (<255.0) | 763 | 67 (8.8) | ref |  | ref |  |  |
|  | Q2 (255.0-344.6) | 763 | 63 (8.3) | 1.00 (0.68-1.45) ^#^ | 0.98 | 0.95 (0.65-1.39) ^#^ | 0.80 |  |
|  | Q3 (344.7-466.9) | 763 | 57 (7.5) | 0.86 (0.58-1.26) ^#^ | 0.43 | 0.82 (0.56-1.21) ^#^ | 0.32 |  |
|  | Q4 (≥467.0) | 763 | 112 (14.7) | 1.85 (1.31-2.59) ^#^ | <0.001 | 1.70 (1.20-2.41) ^#^ | 0.003 | **0.02** |
| **BG-EPVS (severe)** ^††^ | Q1 (<255.0) | 763 | 8 (1.1) | ref |  | ref |  |  |
|  | Q2 (255.0-344.6) | 763 | 5 (0.7) | 0.70 (0.23-2.20) | 0.55 | 0.64 (0.20-2.04) | 0.45 |  |
|  | Q3 (344.7-466.9) | 763 | 9 (1.2) | 1.26 (0.47-3.34) | 0.65 | 1.26 (0.46-3.45) | 0.65 |  |
|  | Q4 (≥467.0) | 763 | 16 (2.1) | 1.96 (0.82-4.72) | 0.13 | 1.90 (0.77-4.71) | 0.17 | 0.30 |

SII, systemic immune-inflammation index (platelet count × neutrophil count/lymphocyte count); WHM, white matter hyperintensities; CMBs, cerebral microbleeds; BG-PVS, perivascular spaces in basal ganglia; cOR, common odds ratio; OR, odds ratio.

^*^ WMH Burden was defined as either (early) confluent deep white matter hyperintensities (Fazekas score 2 or 3) or irregular periventricular white matter hyperintensities extending into the deep white matter (Fazekas score 3); Modified WMH Burden was classified into grade 0: total periventricular + subcortical white matter hyperintensities score 1-2, grade 1: total periventricular + subcortical white matter hyperintensities score 3-4 and grade 2: total periventricular + subcortical white matter hyperintensities score 5-6.

^†^ Presence of CMBs was defined as presence of cerebral microbleeds; CMBs Burden was classified as grade 0: absent, grade 1: 1-4 microbleeds and grade 2: ≥5 microbleeds.

^††^ BG-EPVS (moderate-to-severe) indicated >10 perivascular space in basal ganglia; BG-EPVS (severe) indicated severe (>20) perivascular space in basal ganglia.

^‡^ Model1: multivariable logistic regression model adjusted for covariates: age, sex.

^§^ Model2: multivariable logistic regression model adjusted for covariates: age, sex, body mass index, diabetes, hypertension, total cholesterol, high-density lipoprotein, low density lipoprotein, fasting blood glucose, homocysteine, previous dyslipidemia, previous heart disease, current smoking, current drinking, previous antiplatelet, anticoagulant, antihypertensive, antidiabetic, lipid-lowing drugs use.

^#^ As Modified WMH Burden and CMBs Burden were ordinal categorical variables, ordinal logistic regression model was used and cORs were presented, whereas logistic regression analysis was used and ORs were presented for others.

**TABLE S9. Mendelian randomization** **pleiotropy and heterogeneity analyses for the association of NC with** **CSVD phenotypes.**

| **Phenotypes** | **Egger Intercept** | | **Cochran’s Q statistic in IVW** | |
| --- | --- | --- | --- | --- |
|  | **Intercept (95% CI)** | **P Value** | **Q Value** | **P Value** |
| **WMH volume** | -0.001 (-0.004, 0.002) | 0.72 | 494.93 | 3.42E-06 |
| **Lacunar stroke** | -0.001 (-0.009, 0.006) | 0.71 | 384.76 | 1.12E-05 |
| **Small vessel stroke** | -0.004 (-0.011, 0.003) | 0.28 | 417.05 | 0.003 |
| **Any CMBs** | -0.004 (-0.013, 0.005) | 0.38 | 341.11 | 0.35 |
| **Mixed CMBs** | 0.003 (-0.012, 0.019) | 0.70 | 297.73 | 0.88 |
| **Lobar CMBs** | -0.007 (-0.017, 0.004) | 0.20 | 329.66 | 0.51 |

NC, neutrophil count; CSVD, cerebral small vessel disease; WMH, white matter hyperintensities; CMBs, cerebral microbleeds.

**TABLE S10. Sensitivity analyses of Mendelian randomization for the association of NC with CSVD phenotypes.**

| **Phenotypes** | **MR PRESSO** | | **Weighted Median** | | **MR-egger** | | **Weighted mode** | |
| --- | --- | --- | --- | --- | --- | --- | --- | --- |
|  | **ORs (95% CI)** | **P Value** | **ORs (95% CI)** | **P Value** | **ORs (****95% CI)** | **P Value** | **ORs (95% CI)** | **P Value** |
| **WMH volume** | 0.94 (0.83-1.07) | 0.34 | 0.95 (0.88-1.03) | 0.18 | 1.00 (0.89-1.13) | 0.98 | 0.93 (0.80-1.08) | 0.33 |
| **Lacunar stroke** | **1.48 (1.09**-**2.02)** | **0.01** | **1.26 (1.03**-**1.55)** | **0.03** | 1.27 (0.91-1.78) | 0.16 | 1.22 (0.92-1.61) | 0.17 |
| **Small vessel stroke** | **1.44 (1.06**-**1.95)** | **0.02** | **1.36 (1.11**-**1.66)** | **0.002** | **1.41 (1.03**-**1.92)** | **0.03** | **1.44 (1.08-1.91)** | **0.01** |
| **Any CMBs** | 0.84 (0.57-1.24) | 0.38 | 0.93 (0.71-1.22) | 0.60 | 1.08 (0.73-1.60) | 0.68 | 0.92 (0.59-1.44) | 0.72 |
| **Mixed CMBs** | 0.65 (0.34-1.24) | 0.19 | 0.86 (0.54-1.39) | 0.55 | 0.73 (0.37-1.44) | 0.37 | 0.93 (0.47-1.82) | 0.83 |
| **Lobar CMBs** | 0.90 (0.56-1.44) | 0.66 | 1.04 (0.74-1.46) | 0.83 | 1.25 (0.79-1.99) | 0.34 | 1.09 (0.68-1.75) | 0.72 |

NC, neutrophil count; CSVD, cerebral small vessel disease; WMH, white matter hyperintensities; CMBs, cerebral microbleeds; ORs, odds ratios.

The effect estimates of genetically predicted neutrophil count on CSVD phenotypes were presented as odds ratios (ORs) with their 95% CIs per 1-SD (standard deviation) increment of neutrophil count.
